# Exosomes: A New Pathway for Cancer Drug Resistance

**DOI:** 10.3389/fonc.2021.743556

**Published:** 2021-09-24

**Authors:** Yunbin Zhong, Haibo Li, Peiwen Li, Yong Chen, Mengyao Zhang, Zhendong Yuan, Yufang Zhang, Zhijie Xu, Geng Luo, Yuan Fang, Xu Li

**Affiliations:** ^1^ Hand, Foot Vascular Surgery, Tungwah Hospital to Sun Yet-sen University, Dongguan, China; ^2^ Department of Plastic Surgery, The Third Xiangya Hospital, Central South University, Changsha, China; ^3^ Dermatology Department, The First Hospital of Changsha, Changsha, China; ^4^ Anyang Tumor Hospital, The Fourth Affiliated Hospital of Henan University of Science and Technology, Anyang, China; ^5^ Department of Pathology, Xiangya Hospital, Central South University, Changsha, China; ^6^ Department of Plastic and Reconstructive Surgery, Shanghai Ninth People’s Hospital, Shanghai Jiao Tong University School of Medicine, Shanghai, China

**Keywords:** exosomes, cancer, chemoresistance, drug resistance, tumor microenvironment, signal transmission

## Abstract

Exosomes are extracellular vesicles (EVs) that are secreted into body fluids by multiple cell types and are enriched in bioactive molecules, although their exact contents depend on the cells of origin. Studies have shown that exosomes in the tumor microenvironment affect tumor growth, metastasis and drug resistance by mediating intercellular communication and the transport of specific molecules, although their exact mechanisms of action need to be investigated further. In this review, we have summarized current knowledge on the relationship between tumor drug resistance and exosomes, and have discussed the potential applications of exosomes as diagnostic biomarkers and therapeutic targets.

## Introduction

Exosomes were discovered by Pan et al. while studying the transition of reticulocytes to mature red blood cells (1), and were later defined by Johnstone et al. as vesicles that containing non-essential proteins that are expelled from cells (2) following the fusion of microvesicles with the cell membranes (3). Subsequent studies showed that exosomes are released from blood cells, tumor cells, epithelial cells, mesenchymal stem cells and neuronal cells into the blood, saliva, urine and other body fluids (4–7). Exosomes were first considered to be “garbage bags” that remove waste proteins and metabolites. However, recent studies have shown that the exosomal cargo is biologically active (8) and mediates intercellular communication (9). Several exosomes-enriched proteins, including cytoskeletal proteins, fusion-related proteins, tetrapeptides (CD9, CD63, CD81 and CD82) and membrane connectins (10, 11), as well as oncoproteins (12), have been reported on in recent years. Furthermore, exosomes are also known to transport nucleic acids, such as DNA and coding and non-coding RNAs (13), including micro RNAs (miRNAs) and circular RNAs (circRNAs) (14), that regulate various aspects of tumor development, including immunosuppression, angiogenesis, cell migration and invasion (15–17). Electron microscopy studies have revealed that exosomes measure 50-150 nm in diameter and have a “dish-like” or “cup-shaped” morphology. In addition, several surface exosomal proteins, such as the lysosomal protein Lamp2b, heat shock protein Hsp70 and others (18, 19) that serve as diagnostic markers have also been identified.

Chemotherapeutic drug resistance is a major challenge in cancer treatment. Tumor cells are either naturally resistant to drugs or acquire resistance over the course of treatment (20). Acquired drug resistance is the result of mutations, polymorphisms and splicing variations in genes related to drug metabolism and toxicity (21). A major mechanism of drug resistance in tumor cells is the overexpression of membrane transporters, in particular the ATP binding box (ABC) transporter protein, which rapidly expel drugs and thus reduces their intracellular levels. Furthermore, mutations in drug targets can decrease the efficacy and toxicity of drugs. Interestingly, studies have shown that exposure to chemotherapy drugs significantly increases mutation rates in cancer cells, which may be partially attributed to the activation of pro-survival and anti-apoptotic pathways (22). Furthermore, tumor cells have a higher DNA repair rate compared with normal cells, which neutralizes any DNA damage induced by chemotherapeutic drugs and generates drug-resistant clones. Given that exosomes are involved in the aforementioned signaling pathways, it is important to explore their role in the development of drug resistance (23). In this review, we have summarized the common mechanisms of the genesis of chemoresistance in cancer cells and have also discussed the possible involvement of exosomes.

## Exosomal-Mediated Tumor Resistance

### Exosomes Participate in Tumor Microenvironment Regulation

**Figure 1:**
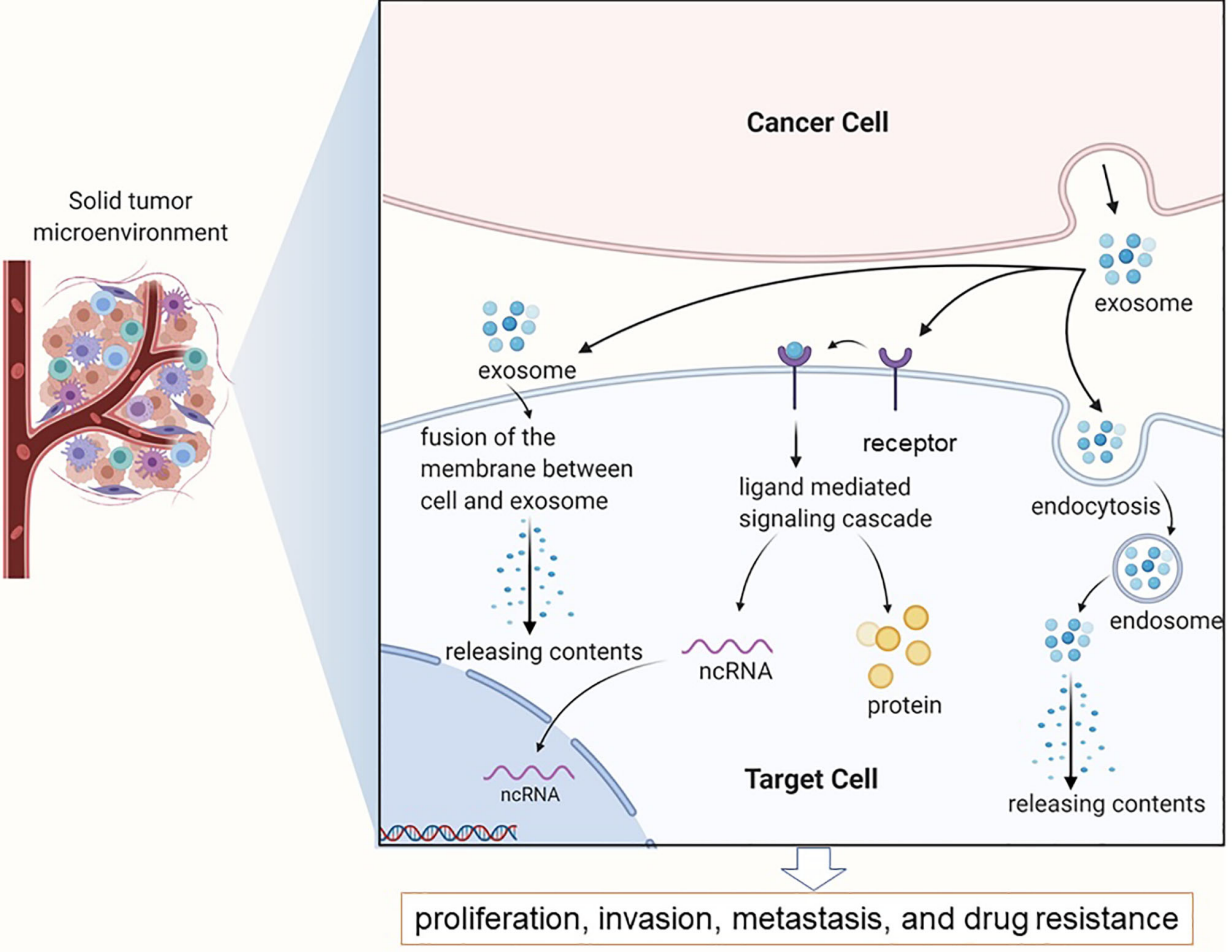
Exosomes in the tumor microenvironment mainly complete information exchange and material transportation between tumor cells.

The tumor microenvironment (TME) is comprised of fibroblasts, stromal cells and the extracellular matrix that aid in the survival, proliferation and metastasis of tumor cells (24, 25). Exosomes mediate cell-to-cell communication in the TME by shuttling signaling molecules, lipids, proteins, nucleic acids and metabolites. In addition, exosomes released from the tumor cells and stromal cells can regulate drug resistance by directly interacting with drug molecules, altering the transcriptome of cancer cells and influencing the immune response (26). The extracellular acidity of tumors markedly affects exosome release by tumors in terms of both the amounts of released exosomes and the make-up of the exosomes (27–30). As shown in **Figure 1** (31), exosomes release active molecules, such as ncRNAs and proteins into target cells following receptor ligand interactions, membrane fusion, as well as puffing, phagocytosis, or receptor-mediated endocytosis, which then regulates tumor cell proliferation, invasion, metastasis and drug resistance (32, 33).

Tumor cells induce adaptive changes in distant organs to create a “pre-metastatic” environment that is conducive to their growth, and the formation of the secondary metastatic foci (34). Zhou et al. showed that exosomes derived from highly metastatic breast cancer cells express high levels of miR-105 (35). Absorption of these exosomes by pulmonary microvascular endothelial cells leads to the significant downregulation of ZO-1, which increases vascular permeability and facilitates the colonization of lung tissues by the tumor cells. Likewise, Yu et al. found that tumor cell-derived exosomes induced the formation of a pre-metastatic niche in the liver of a mouse model of pancreatic cancer, which enhanced metastasis and primary tumor growth. In addition, Costa-Silva et al. found that pancreatic cancer cells release exosomes loaded with macrophage migration inhibitory factors that are absorbed by the hepatic Kupffer cells. This promotes TGF-βD secretion and up-regulates the fibronectin level, and increases the metastasis of tumor cells to the liver. Evidence also indicates that the TME plays a role in drug resistance (36, 37). PGP transported by exosomes can fuse with the plasma membrane of osteosarcoma cells, and P-glycoprotein (P-gp) transporters that enrich tumor cells, resulting in pharmacological desensitization (38). In addition, exosomal P-gp in the serum of prostate cancer patients is a potential biomarker of docetaxel resistance (39).

### Exosomes Participate in Tumor Local Immune Microenvironment Regulation

The TME harbors multiple immune cells including T lymphocytes, neutrophils, NK cells and the tumor-associated macrophages (TAMs). The T cells and TAMs in particular play a significant role in tumor genesis, development and drug resistance, while exosomes in the TME for instrumental in the interactions between immune cells and cancer cells. Exosomes released by the CD8+ T cells contain O-GlcNAc transferase, which upregulates PD-1 in the recipient cells and creates an immunosuppressive environment (40). Previous studies have shown that cancer cell-derived exosomes not only promote anti-tumor immunity but also enhance tumor immune escape (41). Binenbaum et al. demonstrated that when macrophage-derived exosomes expressing miR-365 are internalized by pancreatic ductal adenocarcinoma (PDAC) cells, the tri-phospho-nucleotide pool in cancer cells increases and activates cytidine deaminase, which then inducing resistance to gemcitabine (42).

### Signal Transmission Between Drug-Resistant Cells and Sensitive Cells

Given the highly heterogeneous nature of tumors, individual cells of the same tumor mass differ in their response to chemotherapeutic drugs. Interestingly, chemoresistance or chemosensitivity can be transferred between cells *via* exosomes (see **Table 1**). For instance, exosomes derived from cisplatin-resistant lung cancer cells induced drug resistance in recipient cells *via* miRNA-100-5p, which altered mTOR expression levels (43). Fu X et al. showed that multidrug-resistant liver cells transferred miRNA-32-5p to sensitive cells through exosomes, which then activated phosphatidylinositol kinase *via* the protein kinase B (Akt) pathway and induced drug resistance (44). Compared with chemo-sensitive breast cancer cell lines such as MCF-7, the resistant strains express significantly higher levels of miRNA-222. The exosomes secreted by azithromycin-resistant breast cancer cells can confer drug resistance to sensitive recipient cells *via* miR-222, which is known to regulate cell cycle and apoptosis-related genes (45). Zhang et al. showed that colon cancer cells-derived exosomes promoted cetuximab resistance by downregulating human chromosome 10 phosphatase (PTEN) and increasing phosphorylated Akt levels (46). Furthermore, the exosomes released by gemcitabine-resistant cancer cells delivered miRNA-222-3p to recipient cells through endocytosis, which promote drug resistance and malignant progression by targeting SOCS3 (47). Takahashi et al. found that sorafenib-resistant liver cancer cells expressed high levels of the lncRNA RoR that modulates the TGF-β signaling pathway. Furthermore, the co-culture of sensitive liver cancer cells with lncRNA-RoR exosomes released by drug-resistant cells was able to induce sorafenib resistance (48). Qu et al. found that exosomal lncARSR induced sunitinib resistance in renal cancer cells by sponging miR-34 and miR-449 and upregulating receptor tyrosine kinase (AXL) and c-MET (49).

**Table 1:** Signal transmission between drug-resistant cells and sensitive cells.

Disease	Cells	Drug resistance	Exosomal RNA	Mechanism/pathway	References
Lung cancer		Cisplatin	miR-100-5p	mTOR	(43)
Hepatocellular carcinoma	Bel/5-FU Bel7402	Multidrug resistance	miR-32-5p	PTEN/PI3K/Akt	(44)
Breast cancer	MCF-7	Adriamycin	miR-222		(45)
Colon cancer	RKO Caco-2	Cetuximab		PTEN/Akt	(46)
non-small cell lung cancer	A549-GR	Gemcitabine	miR-222-3p	SOCS3	(47)
Hepatocellular carcinoma	HepG2 PLC-PRF5	Sorafenib	linc-RoR	TGFβ	(48)
Renal Cancer		Sunitinib	lncARSR	miR-34/miR-449 AXL/c-MET	(49)

### Signal Transmission Between Stromal Cells and Tumor Cells

Exosomes derived from stromal cells can also induce drug resistance (**Table 2**). Zheng et al. showed that exosomes derived from M2 macrophages conferred cisplatin resistance to gastric cancer cells through miRNA-21, which inhibited apoptosis by downregulating PTEN and activating the PI3K/Akt signaling pathway (50, 55). Likewise, Ji et al. found that exosomes derived from mesenchymal stem cells (MSCs) induced fluorouracil resistance in gastric cancer cells by activating the CaMKs/Raf/MEK/ERK pathway (51). In addition, exosomes secreted by bone marrow matrix cells induced bortezomib resistance in myeloma cells (52), whereas colon tumors in mice developed resistance to fluorouracil or oxaliplatin in the presence of fibroblast-derived exosomes (53). Boelens et al. found that matrix cells in breast tumors trigger drug resistance in a paracrine manner through exosomal RNAs that activate the NOTCH3 signaling pathway (54).

**Table 2:** Signal transmission between stromal cells and tumor cells.

Disease	Stromal cells	Drug resistance	Exosomal RNA	Mechanism/pathway	References
Gastric cancer	M2 macrophages	Cisplatin	miR-21	PTEN-PI3K- Akt	(50)
Gastric cancer	MSCs	5-fluorouracil		CaM-Ks/Raf/MEK/ERK	(51)
Multiple myeloma	Patients’ peripheral blood	Bortezomib	FFAR1/SP9/HIST1H2BG/ITIH2	mTOR/cAMP/PI3K-Akt	(52)
Colorectal cancer	Fibroblasts	5-fluorouracil Oxaliplatin		Promoted percentage, clonogenicity and tumor growth	(53)
Breast cancer	Stromal cells		5’-triphosphate exoRNA	STAT1/NOTCH3	(54)

As shown through the introduction to this section, exosomes play an important role in various types of transmissions, including the transmission of cisplatin and therapeutic antibodies (56–58). Exosomes with functional enzyme molecules can also be detected in human plasma and may play a pivotal guiding role in cancer progression (59). Exosomes carry molecules with the dual functions of being tracers and therapeutic molecules indicating their diagnostic and therapeutic potential (57). Exosomes are involved in regulating the direction material movement. Recently, researchers have also used exosomes to transfer nano materials (60). Therefore, the potential applications of exosomes are not only limited to its role in the development of tumor drug resistance.

## The Role And Mechanism of Action of Exosomes in the Development of Tumor Resistance

### Regulation of Neoplastic Growth and Metastasis

Cancer stem cells (CSCs) are the source of primary and metastatic tumors, as well as the basis of chemo- and radio-resistance, which leads to tumor recurrence (61). Studies have shown that CSCs often reappear after chemotherapy and express the ATP binding cassette subfamily B member 5 (ABCB5) protein, which mediates multidrug resistance in multiple cancers. The stem cell-derived exosomes express pluripotency-related transcription factors such as Nanog, Oct-4, HOXB4, Rex-1, and Wnt-3, which can endow recipient cells with “stemness” features, such as self-renewal, expansion and differentiation into progenitor cells (62). In addition, exosomes secreted by the cancer-associated fibroblasts (CAFs) express Snail1, which can induce the epithelial-mesenchymal transition of recipient lung cancer cells (63). A recent study showed that tumor cells cultured in a conditioned medium of CAFs showed a higher proliferation rates in the presence of 5-fluorouracil or oxaliplatin, compared with cells grown in normal culture medium (53). Thus, drugs that target the CAFs can potentially sensitize tumor cells to chemotherapy. Furthermore, exosomes secreted by CSCs promote tumor growth and metastasis through paracrine and endocrine modes. The inhibition of exosome secretion can slow clonal expansion and tumor growth (64).

### Expression of Transcription Factors

MiR-210 is expressed at significantly higher levels in colorectal cancer (CRC) tissues compared with that of normal colon tissues, and is associated with an increased level of metastasis. The exosomes secreted by the primary CRC cell line, HCT-8, induced 5-fluorouracil and folate resistance in the chemo-sensitive cells by delivering miR-210, which significantly increased sib levels (65). Non-small cell lung cancer (NSCLC) is usually of one of two types, either chemotherapeutic resistance or tumor rapid metastasis and spread. It is generally believed that this resistance is caused by mesenchymal NSCLC cells, but the mechanism of metastasis is unclear. R. J. et al. suggested that exosomes release by these transformed mesenchymal phenotype cancer cells could induce drug resistance in parental EPC and increased the expression of ZEB1 mRNA in receptor cells (66). Furthermore, T cell-derived exosomes triggered the epithelial mesenchymal transition (EMT) of esophageal cancer cells (67), and the exosomes released from CAFs promoted cancer cell growth and EMT *via* miR-21, miR-378e and miR-143 (68).

### Cell Cycle and Apoptosis

MiR-21 is transferred from resident fat cells or fibroblasts in ovarian tumors to the cancer cells, and can induce drug resistance by inhibiting APAF1-mediated apoptosis (69). In addition, the exosomes secreted by the M2 polarized macrophages can induce cisplatin resistance in gastric cancer cells by directly delivering miR-21 into the recipient cells (50). Furthermore, Her2+ breast cancer cells-derived exosomes can promote trastuzumab resistance *via* lncSNHG14 that targets the Bcl-2/BAX apoptotic pathway (70). The PLX-4720 BRAF inhibitor–resistant melanoma cells were able to activate of PI3K/AKT signaling and inhibition of the MAPK pathway (71). A recent study showed that exosomes containing miR-32-5p induced multi-drug resistance by activating the PI3K/AKT pathway and inhibiting PTEN (44).

### Drug Efflux and Metabolism

The concentration of drugs used for the treatment of cancer cells can be reduced to therapeutically sub-optimal levels by decreasing its permeability through the cell membrane and/or increasing its active efflux. L.V. et al. found that exosomes can efficiently transfer p-glycoprotein (p-gp) from chemo-resistant breast cancer cells to sensitive cells, thus inducing resistance in the latter through increased drug efflux (72). Based on these findings, p-gp was hypothesized to be a drug efflux pump encoded by the ABCB1 gene (73). The experiment conducted by L.V. et al. indicated that when adding MCF-7/DOC that extract of supernatant fluid secretion body (DOC/exobiology) for culture, MCF-7/S resistance can be induced, and to join the MCF-7/S exosomes (S/exobiology) training, MCF-7/S did not acquire resistance. When MCF-7/S and DOC/exo was co-cultured, the level of p-glycoprotein expression raised based on the number of exosomes. Similar to the finding of the above mentioned studies, Ning et al. confirmed that exosomes secreted by MCF7/ADM carried UCH-L1 and p-glycoprotein (74). Taken together, the above mentioned findings indicate that drug-resistant breast cancer cells can secrete exosomes containing p-gp, and confer chemoresistance to the more sensitive recipients. The exosomes secreted by the docetaxel-sensitive DU145 prostate cancer cells (DU145 tax-sen) are enriched in MDR1 (ABCB1), mdr-3, endophilin-a2, and PABP4, which are potential biomarkers of docetaxel resistance (75).

In conclusion, exosomes secreted by tumor related cells can promote tumor growth and metastasis through a variety of action pathways. At the same time, some small RNA molecules can affect cell apoptosis, so as to achieve the effect of drug resistance. If the exosome carrier is no longer small RNA, but a drug, it can induce drug resistance in the process of endocytosis and exocytosis. The ability of exosomes to repair DNA or block the process of transferring mRNA can increase the sensitivity of chemotherapy and radiotherapy and achieve better therapeutic effect.

## The Role of Exosomes in Tumor Drug Resistance

**Figure 2:**
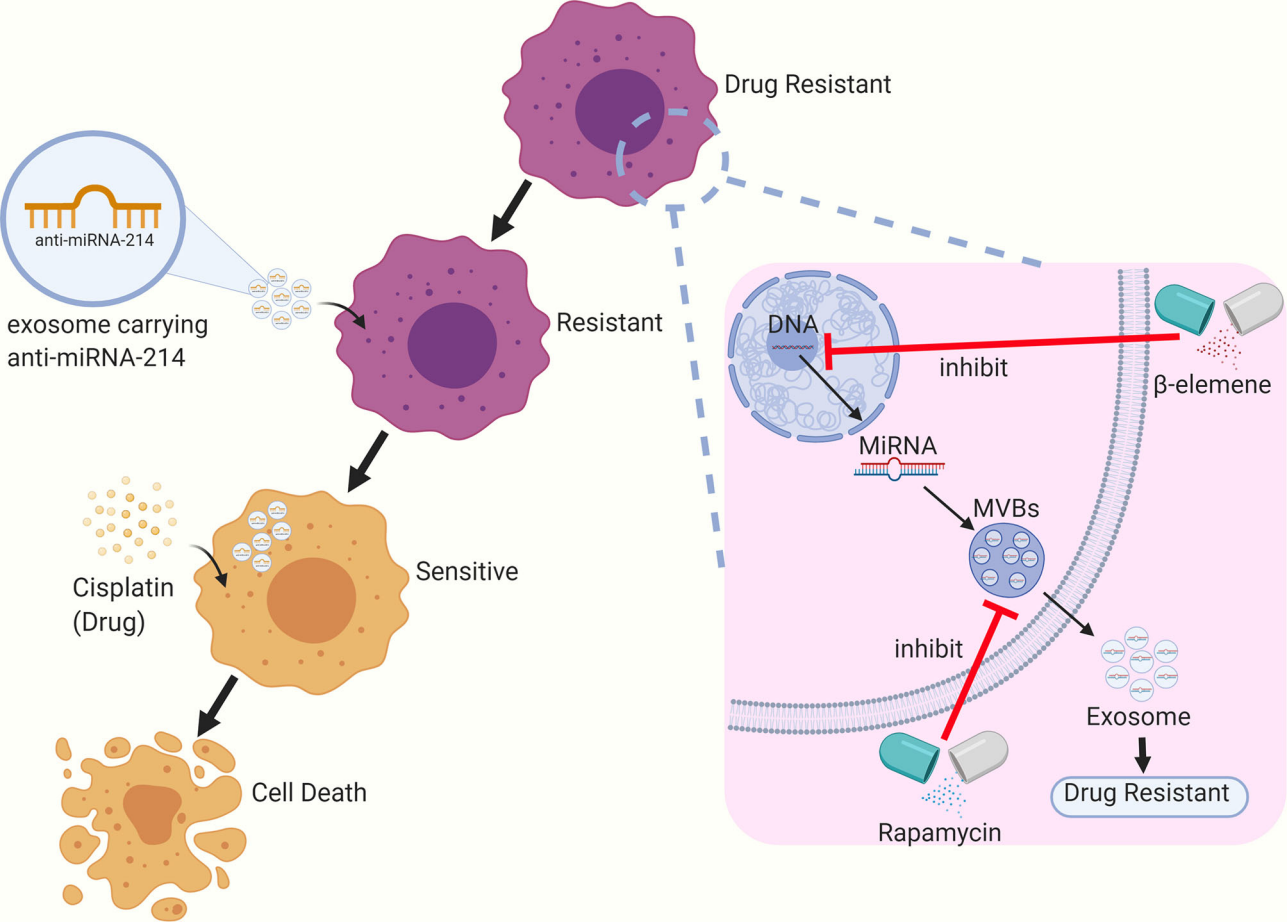
The role of exosomes in the treatment of tumor drug resistance.

Based on the available research, exosomes have shown to be promising nanocarriers that can be used for the reversal of tumor drug resistance. For instance, Wang et al. sensitized cisplatin-resistant gastric cancer cells by directly delivering anti-miRNA-214 to the recipient cells through exosomes (76). Rapamycin and U18666A can sensitize B lymphoma cells to rituximab *via* the inhibition of exosome release by interfering with the synthesis of MVBs and the incorporation of cholesterol into cell membranes. Some researchers have found that β-elemene can act on targeted genes in breast cancer cell lines to alter the expression of resistance-related miRNAs in exosomes, thereby reducing the level of resistance transmission through exosomes and enhancing the sensitivity to chemotherapy (77) (see **Figure 2**).

### Targeting Exosomes to Reverse Chemoresistance

In recent years, several studies have demonstrated that exosomes can be targeted to prevent the development and reverse the chemoresistance of cancer cells. For instance, Cao et al. showed that the neutral sphingomyelinase (NSM) inhibitor, GW4869, sensitized cisplatin-resistant ovarian cancer cells, indicating the therapeutic potential of this novel drug in the recalcitrant cancer patients (78). Furthermore, studies have shown that ketotifen, cannabinol (CBD) and psoralen can sensitize tumor cells to chemotherapeutic drugs by reducing exosome secretion from these cells. Likewise, rhamnose-emodin can reduce exosome secretion from the doxorubicin-resistant breast cancer cells and downregulate the expression of exosomal miRNAs involved in chemoresistance, reversing drug resistance. The exosomes secreted by human umbilical cord mesenchymal cells (hUC-MSC-Exo) can sensitize myelogenous leukemia K562 cells to imatinib by activating the caspase signaling pathway (79). Therefore, the combination of imatinib and hUC-MSC-Exo is a promising therapeutic strategy against chronic myelogenous leukemia (CML) (79). Li et al. found that the exosome-specific miRNA-770 reversed doxorubicin resistance in triple negative breast cancer (TNBC) cells by regulating apoptosis pathways and the TME (80). In addition, Akt inhibitors could reverse the chemoresistance of sensitive cancer cells induced by exosomes derived from the drug-resistant cells (81). Wang B. et al. found that the IC50 of cisplatin in chemo-sensitive TNBC cells increased 2.24 times after being co-cultured with a chemo-resistant cell line but decreased upon treatment with the compound, Yiqi (82).

### Exosome and Tumor Chemotherapy Resistance Markers

Exosomes can be isolated from various biological fluids, such as blood, urine and saliva. The blood of healthy individuals may contain over 2000 trillion exosomes, whereas that of cancer patients contains 4000 trillion exosomes. Thus, tumor cells may produce and secrete more exosomes compared with normal cells, and can be used as potential diagnostic biomarkers (83). Yuwen et al. correlated lower serum levels of exosomal miRNA-146a-5p in patients with advanced NSCLC with a higher recurrence rate (84). In addition, serum exosomal miRNA-146a-5p is a novel biomarker that can be used to predict and monitor cisplatin resistance in NSCLC patients. Likewise, the serum exosomes enrichment of in TAG72 indicates a high probability of 5-FU resistance in CRC cells (85), and that of exosomal miRNA-222-3p predicts gemcitabine sensitivity in NSCLC patients (47). TRPC5 expression in breast cancer tissues and patient response to chemotherapy are significantly correlated with the level of cirExo-TRPC5 in peripheral blood. Since cirExo-TRPCS levels increase after chemotherapy, it can be used as a promising biomarker for the image-based detection of chemo-resistance (86). High expression of GSTP1 in circulating exosomes may indicate an increase of chemo-resistance. A clinical study showed that the level of miRNA-151a in cerebrospinal fluid (CSF)-derived exosomes reflects potential chemo-resistance of glioblastoma multiforme (GBM) patients (87). In addition, Leukemia-derived exosomes induced IL-8 production in bone marrow stromal cells, which can protect acute myeloid leukemia cells from chemotherapy drug induced apoptosis (88). The content of exosomes secreted by tumor cells changes along with the level of cellular stress induced by anticancer therapy, which leads to the metastasis of drug resistant phenotypes in breast cancer (72, 82). This leads to the transfer of drug resistance mediated by exosomes between drug resistant cells and sensitive breast cancer cells (89). Moreover, the selective isolation of circulating subsets of exosomes enriched in tumor sources could effectively improve the sensitivity and specificity of detection (90).

Then, according to the above, we boldly speculate that in the future, it can be separated from various human biological liquids, such as blood, urine and salivary blood, which could directly detect some disease-related exosomes bio-markers, so as to predict the condition and curative effect of patients at this stage. Then, some drugs are used to target the corresponding exosomes to reverse the drug resistance. This may be a simple, convenient and universal treatment in the future.

## Conclusion and Prospects

Exosomes mediate intercellular communication in the TME, and can induce drug resistance in tumor cells by transferring specific mRNAs, ncRNAs or proteins (91). The differential expression of these molecules in exosomes are useful clinical markers of tumor drug resistance (92). Therefore, there is a clear need to further elucidate the role of exosomes in tumor drug resistance to improve prognostic prediction and therapeutic efficacy.

Exosomes in the TME have increasingly been identified as the vectors of oncogenesis, drug resistance and metastasis, although the exact pathways and mechanisms involved are still unclear. A deeper understanding of these mechanisms will provide new insights into tumor heterogeneity, and significant differences in the chemotherapeutic responses of individual cancer patients. Furthermore, exosomes are also promising nanocarriers for the targeted delivery of drugs to tumor cells. Given that the prognosis of cancer patients is closely associated with natural or acquired chemo-resistance of the tumor cells, early identification of recalcitrant patients can help formulate individualized optimal treatment regimens. Tumor patients have a higher level of plasmatic exosomes compared with healthy individuals, independently of tumor histology. This difference indicates that exosomes released by tumors during chemotherapy may deliver cytotoxic drugs to healthy organs, thus inducing harmful effects (29, 93, 94). Several ncRNAs that can regulate tumor cell proliferation, metastasis, chemoresistance and recurrence have been identified in recent years. Circulating exosomes that contain cancer-specific miRNAs and lncRNAs are promising diagnostic/prognostic biomarkers and therapeutic targets in cancer, although their underlying mechanisms of action remain to be clarified.

## Author Contributions

YF and XL performed the research. HL, YF, and XL designed the research study. PL and YFZ contributed essential reagents or tools. YF, HL, and YC helped to analyze the data. XL wrote the article. YF, ZY, and GL revised the article. ZX modified the language. YBZ provided financial assistance. All authors contributed to the article and approved the submitted version.

## Funding

This work was supported by a grant from National Major Science and Technology Projects of Hunan Province (2020JJ4633).

## Conflict of Interest

The authors declare that the research was conducted in the absence of any commercial or financial relationships that could be construed as a potential conflict of interest.

## Publisher’s Note

All claims expressed in this article are solely those of the authors and do not necessarily represent those of their affiliated organizations, or those of the publisher, the editors and the reviewers. Any product that may be evaluated in this article, or claim that may be made by its manufacturer, is not guaranteed or endorsed by the publisher.
